# Preoperative Delays in the Treatment of DCIS and the Associated Incidence of Invasive Breast Cancer

**DOI:** 10.1245/s10434-019-07844-4

**Published:** 2019-09-27

**Authors:** William H. Ward, Lyudmila DeMora, Elizabeth Handorf, Elin R. Sigurdson, Eric A. Ross, John M. Daly, Allison A. Aggon, Richard J. Bleicher

**Affiliations:** 1 Department of Surgery, Naval Medical Center, Portsmouth, VA USA; 2 Biostatistics and Bioinformatics Facility, Fox Chase Cancer Center, Philadelphia, PA USA; 3 Department of Surgical Oncology, Fox Chase Cancer Center, Philadelphia, PA USA

## Abstract

**Background:**

Although treatment delays have been associated with survival impairment for invasive breast cancer, this has not been thoroughly investigated for ductal carcinoma in situ (DCIS). With trials underway to assess whether DCIS can remain unresected, this study was performed to determine whether longer times to surgery are associated with survival impairment or increased invasion.

**Methods:**

A population-based study of prospectively collected national data derived from women with a clinical diagnosis of DCIS between 2004 and 2014 was conducted using the National Cancer Database. Overall survival (OS) and presence of invasion were assessed as functions of time by evaluating five intervals (≤ 30, 31–60, 61–90, 91–120, 121–365 days) between diagnosis and surgery. Subset analyses assessed those having pathologic DCIS versus invasive cancer on final pathology.

**Results:**

Among 140,615 clinical DCIS patients, 123,947 had pathologic diagnosis of DCIS and 16,668 had invasive ductal carcinoma. For all patients, 5-year OS was 95.8% and unadjusted median delay from diagnosis to surgery was 38 days. With each delay interval increase, added relative risk of death was 7.4% (HR 1.07; 95% CI 1.05–1.10; *P* < 0.001). On final pathology, 5-year OS for noninvasive patients was 96.0% (95% CI 95.9–96.1%) versus 94.9% (95% CI 94.6–95.3%) for invasive patients. Increasing delay to surgery was an independent predictor of invasion (OR 1.13; 95% CI 1.11–1.15; *P* < 0.001).

**Conclusions:**

Despite excellent OS for invasive and noninvasive cohorts, invasion was seen more frequently as delay increased. This suggests that DCIS trials evaluating nonoperative management, which represents infinite delay, require long term follow up to ensure outcomes are not compromised.

**Electronic supplementary material:**

The online version of this article (10.1245/s10434-019-07844-4) contains supplementary material, which is available to authorized users.

Ductal carcinoma in situ (DCIS) is a premalignant lesion composed of malignant mammary ductal epithelial cells that have not yet invaded the basement membrane, whose standard interventions have included lumpectomy and radiotherapy or total mastectomy alone, followed by endocrine therapy.^1^–^5^ Defined as American Joint Commission of Cancer (AJCC) Stage 0, patient outcomes following standard treatment are excellent, with 5-year survival typically > 95%.^2^,^6^–^8^

Recently, therapies for DCIS have been scrutinized, as current data suggest that < 50% of afflicted patients will develop invasive cancer without treatment.^1^,^2^,^8^ Additionally, although DCIS detection has increased over recent decades secondary to mammographic improvements, advanced stage distributions have not correspondingly declined, suggesting that screening may detect some DCIS that would remain subclinical.^2^,^4^,^9^–^13^ Many have consequently questioned whether current DCIS paradigms constitute gross overtreatment, exposing thousands of women to therapy-related morbidity unnecessarily.^3^,^4^,^7^,^14^,^15^

Data are limited about intervals between diagnosis and surgery for women undergoing operative resection of DCIS as there is for invasive disease.^16^,^17^ Although we have found decreases in both disease-free and overall survival (OS) with each 30-day delay between histologic diagnosis and definitive surgery for early stage breast carcinoma, data regarding delays to surgery in DCIS is extremely limited.^16^,^18^ This is relevant today, as quality measures examining delay intervals are being considered by accreditation bodies, and ongoing trials are evaluating nonoperative management, which represents infinite “delay” to surgery.^19^,^20^

By examining a large population-based dataset, it is possible to achieve sufficient power to investigate delays in DCIS, elucidating its behavior in two ways. The first investigates whether there is a delay-dependent decline in survival for clinical DCIS that is still pure DCIS on final pathology. The second determines the risk of invasion with increasing delay for those initially staged as DCIS but found on final pathology to have invasion. DCIS lesions that develop and do not develop invasion are cohorts with differing risks that we need to distinguish to clarify which lesions should be aggressively treated and which can be observed.

## Materials and Methods

This analysis of the National Cancer Database (NCDB) is exempt per the Fox Chase Cancer Center Institutional Review Board, and permission was granted from the American College of Surgeons. This is the only dataset of its size for the United States containing the clinical and pathologic staging fields required for analysis. Time intervals between diagnosis and surgery were analyzed continuously, but categorized in 30-day increments with the 121 to 365 interval collapsed into 1 group to maintain sufficient power; thus, intervals were assigned as ≤ 30, 31–60, 61–90, 91–120, and 121–365 days. Date of “definitive surgery” was used as the operative date unless this differed from “first surgery” (28.0% of patients), where the latter was utilized as the operative date. A sensitivity analysis for survival was performed by removing patients with > 1 surgery date. To afford a uniform starting point for all patients, time from surgery was used for OS, and a sensitivity analysis calculating survival from time of diagnosis was also performed.

The cohort was limited to women with Clinical Stage 0 disease. Patients were included if DCIS was their first malignancy, lumpectomy or mastectomy was performed, and diagnosis and any treatment were completed at the reporting facility. Patients transferring facilities between diagnosis and treatment were identified as having a “transfer of care”.^21^ To identify postoperative patients with DCIS or invasive ductal histology, the 2015 ICD-O-3 SEER Site/Histology Validation List was used.^22^ eTable 1 lists included histology codes.

**Fig. 1:**
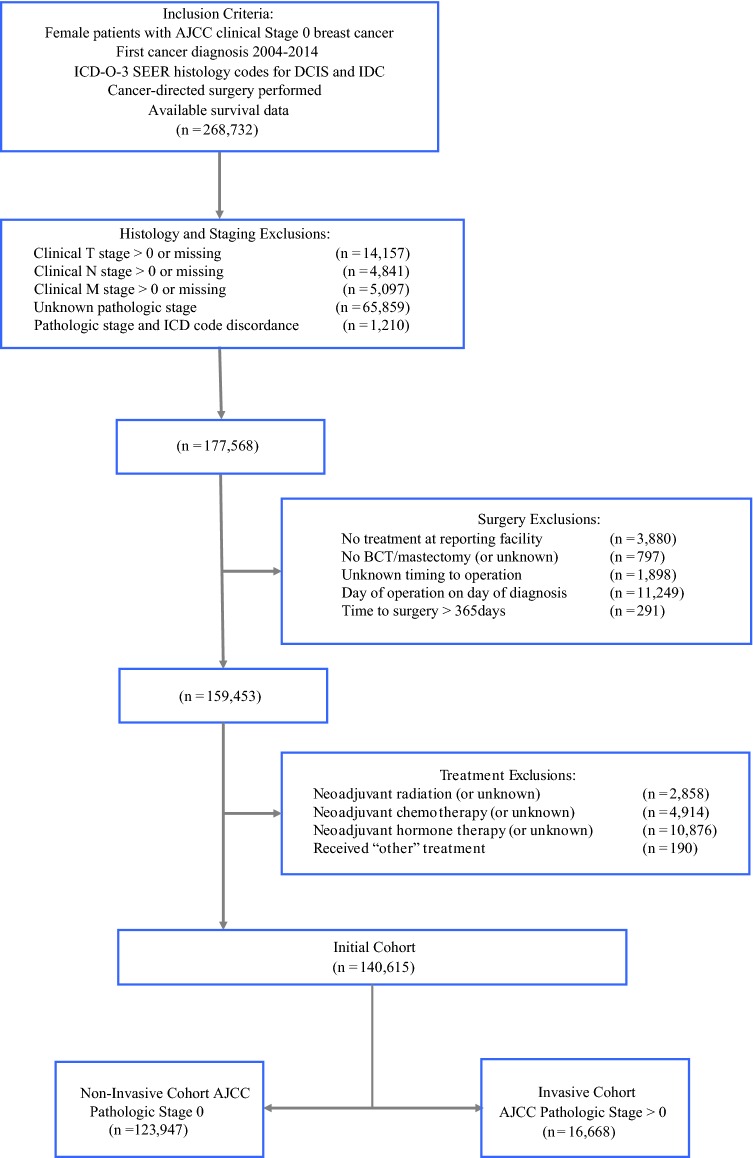
Cohort exclusion criteria. Numbers represent remaining patients after that set of exclusions. *AJCC* American Joint Committee on Cancer; *BCT* breast conservation therapy; *DCIS* ductal carcinoma in situ; *IDC* invasive ductal carcinoma

Patients having conflicts between ICD-O-3 histologic codes versus pathologic stage were excluded, as well as patients whose stage, diagnosis method, treatment order, or survival status was unknown. Women without surgery, having unknown surgical status, time to surgery > 1 year or unknown, and treatment not conducted at the reporting facility also were excluded (Fig. 1).

Within the initial cohort, AJCC Pathologic Stage indicated noninvasive or invasive disease. The noninvasive cohort refers to patients having DCIS on biopsy that also was purely DCIS on final excision. The invasive cohort refers to patients who had DCIS on initial biopsy, but were found to have any extent of invasive ductal carcinoma on final excision. Pathologic diagnoses that did not include ductal histology or subtypes were excluded to remove incidentally found additional primaries, as were those with incomplete pathologic data and staging. Due to space constraints, statistical methods are detailed in the online supplement.

## Results

The 140,615 patients analyzed overall had a median follow up of 57.7 months, and 47.5% of patients had ≥ 60 months of follow-up. Mean (SD) patient age was 58.6 (11.9) years, ranging 18–90. Postoperatively, 123,947 (88.1%) remained pathologically DCIS, whereas 16,668 (11.9%) were found to have invasive disease (Fig. 1).

### Overall Survival

Five-year OS was 95.8% (95% CI 95.7–96.0%). Following adjustment, greater time to surgery (TTS), with multiple other factors, was independently associated with poorer survival (eTable 2). A total of 39,364 (28.0%) patients had > 1 surgery date recorded. A sensitivity analysis (eTable 3) excluding these women from the adjusted analysis found surgical delay significantly associated with OS (HR 1.12; 95% CI 1.09–1.16; *P* < 0.0001).

**Fig. 2:**
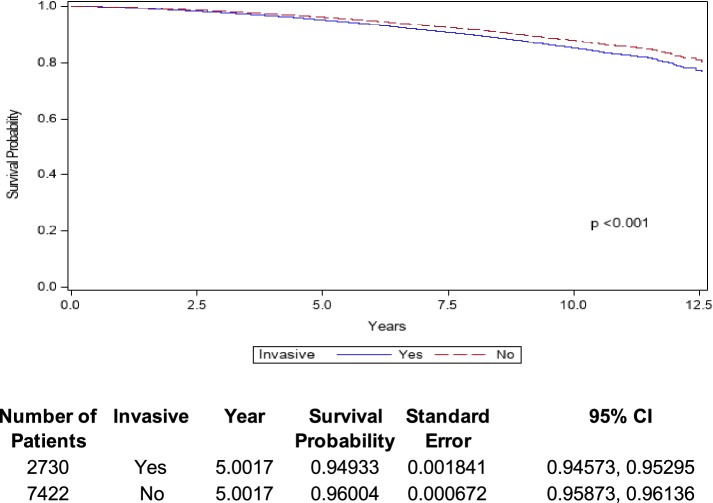
Overall survival of DCIS patients with invasive (blue line) and noninvasive (red line) disease identified on final pathology. *CI* confidence interval; *DCIS* ductal carcinoma in situ

There were 123,947 patients analyzed after excluding those with invasive disease. Mean (SD) age for these patients was 58.7 (11.9) years (range 18–90); 7422 (6.0%) patients died, and adjusted 5-year OS was 96.0% (Fig. 2). Adjusted associations between analyzed variables and OS in patients with noninvasive disease are shown in eTable 4.

Among the 16,668 patients found postoperatively to have invasion, mean (SD) age was 57.3 (12.4) years (range 19–90). Within this cohort, 2730 (16.4%) patients died and the adjusted 5-year OS was 94.9% (Fig. 2). eTable 5 demonstrates the adjusted associations between analyzed variables and OS in patients with invasion.

### Delay to Surgery

Unadjusted median delay from diagnosis to surgery was 38 (IQR: 24–58) days. Patients with intervals of ≤ 30, 31–60, 61–90, 91–120, 121–180, 181–240, and 241–365 days accounted for 37.6%, 38.9%, 14.6%, 5.2%, 2.8%, 0.6%, and 0.3% of the total number, respectively. Patient characteristics are summarized by preoperative interval in eTable 6. After adjustment, characteristics independently associated with increased TTS included Black and Asian race, Hispanic ethnicity, lack of high school diploma, metropolitan setting, greater treating facility distance, transfers of care, and increased Charlson comorbidity score, among others (Table 1). Added risk of death from all causes for each 30-day interval delay increase was 7.4% (HR 1.07; 95% CI 1.05–1.10; *P* < 0.0001) for the entire cohort. Survival point estimates by delay group are listed in eFig. 1 and eTable 7.

**Table 1:** Multivariable adjusted associations between patient characteristics and median delay to surgery

Characteristic	*N* (%)	Median delay (95% CI)	*P*
*Age* (*years*)			
< 50	35,662 (25.4)	40.45933 (40.09947, 40.81920)	
50–59	40,490 (28.8)	39.15395 (38.85004, 39.45785)	< 0.001
60–69	37,081 (26.4)	38.68148 (38.39365, 38.96932)	< 0.001
≥ 70	27,382 (19.5)	37.55041 (37.15215, 37.94867)	< 0.001
*Race*			
White	115,163 (81.9)	38.10018 (37.93171, 38.26865)	
Black	17,042 (12.1)	44.72110 (44.20095, 45.24124)	< 0.001
Asian	5769 (4.1)	40.91739 (40.00678, 41.82800)	< 0.001
Other/unknown	2641 (1.9)	39.69614 (38.61258, 40.77969)	0.005
*Ethnicity*			
Non-hispanic	126,904 (90.2)	38.84077 (38.67952, 39.00202)	
Hispanic	7016 (5.0)	43.74860 (42.84907, 44.64812)	< 0.001
Unknown	6695 (4.8)	38.05353 (37.43627, 38.67080)	0.015
*Insurance*			
Medicaid	6245 (4.4)	45.80981 (44.92331, 46.69630)	
Medicare	41,365 (29.4)	38.84621 (38.49211, 39.20031)	< .001
Uninsured	2127 (1.5)	44.97280 (43.32993, 46.61567)	0.371
Government	1499 (1.1)	40.85378 (39.23582, 42.47175)	< 0.001
Private	87,763 (62.4)	38.43473 (38.22994, 38.63952)	< 0.001
Unknown	1616 (1.1)	41.92920 (40.00933, 43.84906)	< 0.001
Education*			
> 21%	17,870 (12.7)	40.92560 (40.40086, 41.45033)	
13–20.9%	31,550 (22.4)	39.49544 (39.15450, 39.83639)	< 0.001
7–12.9%	46,629 (33.2)	39.16427 (38.91023, 39.41832)	< 0.001
< 7%	43,912 (31.2)	37.92761 (37.60052, 38.25470)	< 0.001
Missing	654 (0.5)	33.13101 (26.41113, 39.85090)	0.025
*Annual income*			
< $38,000	18,831 (13.4)	37.83237 (37.34633, 38.31842)	
$38,000–$47,999	27,816 (19.8)	38.53361 (38.18661, 38.88061)	0.010
$48,000–$62,999	36,940 (26.3)	38.96734 (38.66033, 39.27435)	< 0.001
≥ $63,000	56,337 (40.1)	39.72927 (39.41876, 40.03977)	< 0.001
Missing	691 (0.5)	41.68427 (35.49692, 47.87162)	0.225
*Setting*			
Large metropolitan	79,489 (56.5)	40.58126 (40.36052, 40.80200)	
Small metropolitan	40,922 (29.1)	37.45615 (37.18598, 37.72633)	< 0.001
Suburban	10,438 (7.4)	35.87115 (35.29576, 36.44654)	< 0.001
Rural	5869 (4.2)	33.77957 (33.06795, 34.49119)	< 0.001
Unknown	3897 (2.8)	40.93846 (40.02934, 41.84758)	0.453
*Distance to treatment facility* (*miles*)			
≤ 10	78,606 (55.9)	39.07204 (38.85998, 39.28410)	
11–20	33,342 (23.7)	38.33902 (38.03820, 38.63984)	< 0.001
21–40	16,577 (11.8)	38.51943 (38.05730, 38.98157)	0.033
> 40	11,360 (8.1)	41.64152 (41.03239, 42.25065)	< 0.001
Unknown	730 (0.5)	40.51603 (37.60348, 43.42858)	0.334
*Transfer of care*			
No	86,170 (61.3)	37.54626 (37.35774, 37.73478)	
Yes	39,602 (28.2)	42.89649 (42.55887, 43.23410)	< 0.001
Unknown	14,843 (10.6)	37.49980 (37.09325, 37.90635)	0.837
*Treatment facility annual volume*			
0–17 patients (1st quartile)	8131 (5.8)	36.38818 (35.79152, 36.98483)	
18–34 patients (2nd quartile)	18,054 (12.8)	36.89268 (36.48508, 37.30028)	0.156
35–67 patients (3rd quartile)	35,074 (24.9)	38.30192 (38.01514, 38.58870)	< 0.001
> 67 patients (4th quartile)	79356 (56.4)	40.14092 (39.93916, 40.34269)	< 0.001
*Charlson Comorbidity Index*			
0	120,676 (85.8)	38.97912 (38.81081, 39.14743)	
1	16,780 (11.9)	39.19972 (38.75693, 39.64252)	0.354
2	2659 (1.9)	40.20887 (39.13911, 41.27863)	0.027
≥ 3	500 (0.4)	44.45383 (42.02711, 46.88056)	< 0.001
*Grade*			
Well differentiated	17,357 (12.3)	39.70864 (39.32554, 40.09175)	
Moderately differentiated	47,300 (33.6)	39.57077 (39.31976, 39.82178)	0.547
Poorly differentiated	47,039 (33.5)	38.43289 (38.16999, 38.69579)	< 0.001
Undifferentiated/anaplastic	3684 (2.6)	38.67013 (37.56746, 39.77279)	0.074
Unknown	25,235 (17.9)	38.81639 (38.46441, 39.16837)	0.001
*Surgery type*			
Breast conservation	89,156 (63.4)	34.06873 (33.85780, 34.27965)	
Mastectomy	24,905 (17.7)	42.65004 (42.18069, 43.11938)	< 0.001
Mastectomy with reconstruction	26,554 (18.9)	52.38859 (51.89025, 52.88693)	< 0.001
*Receipt of radiation*			
Yes	70,069 (49.8)	38.59995 (38.34745, 38.85245)	
No	70546 (50.2)	39.49335 (39.24647, 39.74022)	< 0.001
*Receipt of endocrine therapy*			
Yes	54,734 (38.9)	38.41799 (38.17619, 38.65979)	
No	85,881 (61.1)	39.44978 (39.25416, 39.64541)	< 0.001
*Year of diagnosis*			
2004	4758 (3.4)	34.67071 (33.92166, 35.41976)	
2005	5318 (3.8)	35.42238 (34.62557, 36.21918)	0.175
2006	6238 (4.4)	35.81412 (35.11195, 36.51630)	0.029
2007	7444 (5.3)	38.54941 (37.88847, 39.21035)	< 0.001
2008	12,363 (8.8)	39.08158 (38.55450, 39.60865)	< 0.001
2009	14,713 (10.5)	38.80714 (38.36663, 39.24766)	< 0.001
2010	15,636 (11.1)	38.51970 (38.06289, 38.97650)	< 0.001
2011	16,954 (12.1)	38.85152 (38.44292, 39.26013)	< 0.001
2012	18,320 (13.0)	39.64301 (39.25723, 40.02878)	< 0.001
2013	19,626 (14.0)	40.41720 (40.03274, 40.80166)	< 0.001
2014	19,245 (13.7)	41.17652 (40.76929, 41.58375)	< 0.001

*CI* confidence interval; *OR* odds ratio; *Ref* reference group

*Percent of adults without a high school diploma by zip code

Among those without invasion, individuals with intervals of ≤ 30, 31–60, 61–90, 91–120, 121–180, 181–240, and 241–365 days accounted for 38.7%, 38.6%, 14.1%, 5.0%, 2.6%, 0.6%, and 0.3% of the cohort, respectively. The added risk of death from all causes for each 30-day interval increase in delay among the noninvasive cohort was 7.3% (HR 1.07; 95% CI 1.05–1.10; *P* < 0.0001) (eTable 4).

Among the invasive patients, the progressive delay categories accounted for 28.8%, 41.0%, 18.5%, 6.8%, 3.8%, 0.7%, and 0.4% of the cohort, respectively. The added risk of death from all causes for each 30-day interval increase in delay among the invasive cohort was 6.8% (HR 1.07; 95% CI 1.01–1.13; *P* = 0.0306; eTable 5).

### Invasion

**Fig. 3:**
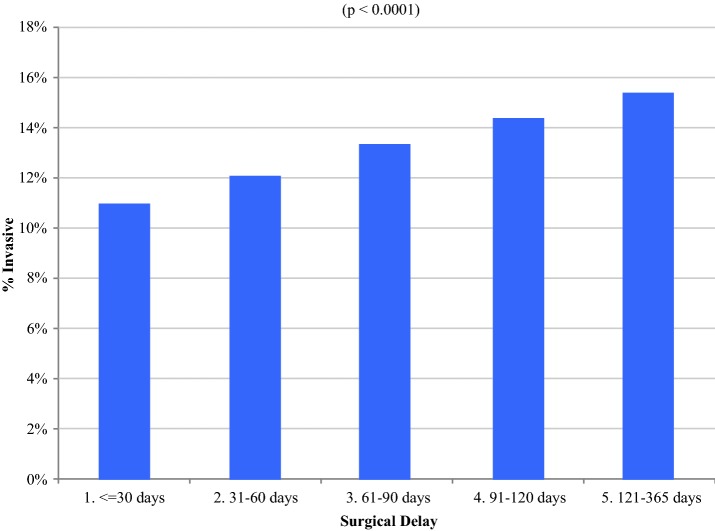
Adjusted proportion of invasion by delay interval. Bars represent the percentage of patients with invasive disease as time to surgery increases (*P* < 0.0001)

As shown in Fig. 3 and Table 2, invasive cancer was increasingly found with greater delay, as well as associated with insurance status, facility distance and volume, transfers of care, year of diagnosis, comorbidities, grade, and ER status. After adjustment, increasing delay to surgery in the entire cohort was an independent predictor of invasion (OR 1.13; 95% CI 1.10–1.15; *P* < 0.001). Among patients with postoperative diagnosis of invasive disease, median invasion measured 5.0 mm (range 0–480). Additional independent predictors of invasion are elaborated in Table 2. A sensitivity analysis in which women having > 1 surgery date were excluded from the adjusted analysis (eTable 8) found that increasing delay to surgery remained an independent predictor of invasion (OR 1.12 per month delay; 95% CI 1.10–1.15; *P* < 0.001). There was no significant difference in the survival effect from delay between women with or without invasion on pathology (*P* = 0.507).

**Table 2:** Multivariable adjusted associations between patient characteristics and invasion

Characteristic	*N* (%)	OR (95% CI)	*P*
Delay from diagnosis to surgery (30-day interval)^§^	140,615 (100)	1.129 (1.110, 1.148)	< 0.0001
*Age* (*years*)^§^			
< 50	35,662 (25.4)	Ref	< 0.0001
50–59	40,490 (28.8)	0.807 (0.773, 0.842)	
60–69	37,081 (26.4)	0.761 (0.724, 0.800)	
≥ 70	27,382 (19.5)	0.737 (0.689, 0.787)	
*Race*			
White	115,163 (81.9)	Ref	0.1205
Black	17,042 (12.1)	1.021 (0.955, 1.091)	
Asian	5769 (4.1)	0.987 (0.895, 1.088)	
Other/unknown	2641 (1.9)	0.850 (0.739, 0.977)	
*Ethnicity*			
Non-hispanic	126,904 (90.2)	Ref	0.0898
Hispanic	7016 (5.0)	1.020 (0.934, 1.114)	
Unknown	6695 (4.8)	0.874 (0.767, 0.995)	
*Insurance*			
Private	87,763 (62.4)	Ref	0.0150
Medicaid	6245 (4.4)	1.135 (1.047, 1.230)	
Medicare	41,365 (29.4)	0.974 (0.922, 1.030)	
Uninsured	2127 (1.5)	1.026 (0.891, 1.182)	
Government	1499 (1.1)	0.989 (0.847, 1.154)	
Unknown	1616 (1.1)	0.780 (0.606, 1.003)	
Education*			
> 21%	17,870 12.7%	Ref	0.1789
13–20.9%	31,550 22.4%	0.962 (0.893, 1.037)	
7–12.9%	46,629 33.2%	1.017 (0.937, 1.105)	
< 7%	43912 31.2%	1.056 (0.957, 1.164)	
Missing	654 0.5%	0.795 (0.275, 2.300)	
*Annual income*			
< $38,000	18,831 (13.4)	Ref	0.1761
$38,000–$47,999	27,816 (19.8)	1.083 (1.004, 1.168)	
$48,000–$62,999	36,940 (26.3)	1.086 (0.997, 1.183)	
≥ $63,000	56,337 (40.1)	1.064 (0.954, 1.186)	
Missing	691 (0.5)	1.704 (0.700, 4.148)	
*Setting*			
Large metropolitan	79,489 (56.5)	Ref	0.0949
Small metropolitan	40,922 (29.1)	0.912 (0.839, 0.991)	
Suburban	10,438 (7.4)	0.963 (0.864, 1.075)	
Rural	5869 (4.2)	0.912 (0.803, 1.035)	
Unknown	3897 (2.8)	1.071 (0.940, 1.220)	
*Distance to treatment facility* (*miles*)			
≤ 10	78,606 (55.9)	Ref	0.0008
11–20	33,342 (23.7)	1.010 (0.964, 1.058)	
21–40	16,577 (11.8)	1.098 (1.021, 1.181)	
> 40	11,360 (8.1)	1.243 (1.133, 1.363)	
Unknown	730 (0.5)	0.668 (0.349, 1.279)	
*Transfer of care*			
No	86,170 (61.3)	Ref	< 0.0001
Yes	39,602 (28.2)	1.234 (1.159, 1.315)	
Unknown	14,843 (10.6)	1.079 (0.995, 1.170)	
*Treatment facility annual volume*			
0–17 patients (1st quartile)	8131 (5.8)	Ref	0.0002
18–34 patients (2nd quartile)	18,054 (12.8)	1.077 (0.944, 1.229)	
35–67 patients (3rd quartile)	35,074 (24.9)	1.119 (0.983, 1.274)	
> 67 patients (4th quartile)	79,356 (56.4)	1.268 (1.122, 1.434)	
Year of diagnosis^§^	140,615 (100)	1.052 (1.038, 1.065)	< 0.001
*Charlson Comorbidity Index*			
0	120,676 (85.8)	Ref	< 0.001
1	16,780 (11.9)	1.133 (1.076, 1.193)	
2	2659 (1.9)	1.279 (1.139, 1.437)	
≥ 3	500 (0.4)	1.181 (0.896, 1.556)	
*Grade*			
Well differentiated	17,357 (12.3)	Ref	< 0.001
Moderately differentiated	47,300 (33.6)	0.534 (0.505, 0.566)	
Poorly differentiated	47,039 (33.5)	0.252 (0.234, 0.271)	
Undifferentiated/anaplastic	3684 (2.6)	0.083 (0.065, 0.105)	
Unknown	25,235 (17.9)	0.354 (0.308, 0.408)	
*Estrogen receptor status*			
Negative	19,836 (14.1)	Ref	< 0.001
Positive	107,305 (76.3)	0.386 (0.366, 0.407)	
Unknown	13,474 (9.6)	0.104 (0.090, 0.121)	

*CI* confidence interval; *OR* odds ratio; *Ref* reference group

*Percent of adults without a high school diploma by zip code

^§^Continuous variable

## Discussion

Before 1980, DCIS was a rare diagnosis, comprising < 5% of identified breast cancers.^23^ Imaging and pathology advances have since increased the diagnosed frequency of DCIS, which now accounts for 25% of breast cancers in the United States.^9^ There has been evolution in treatment, which originally considered DCIS to be an expectant precursor to invasive disease.^9^,^24^,^25^ However, recent reports suggest that a significant number of in situ lesions are overtreated and may remain noninvasive and subclinical if observed. To our knowledge, this is the first investigation examining the association between delays to surgery for DCIS and the presence of invasive disease, and OS, using a large, national, dataset while controlling for confounders through propensity weighting.

As expected, our data confirm that the vast majority of patients with DCIS who undergo surgical extirpation do so in less than 2 months (eFig. 2). We hypothesized that no outcome differences would be observed with longer times to surgery but found that surgical delay was indeed associated with lower OS, and as delay increased, invasion was increasingly found upon excision.

Greater risk of invasion with advancing delay in treating DCIS may be consistent with the fact that at least some DCIS is considered to be a precursor through which neoplastic ductal cells are present but have not yet violated the basement membrane. It should be noted, however, that with any study where DCIS is diagnosed on core biopsy, sampling error for the presence of invasion can occur, and time of transition from DCIS to invasive disease is impossible to determine. Our group previously demonstrated that women with early-stage breast cancer have worse outcomes as TTS increases, suggesting that sufficient time allows tumor growth and spread, conferring poorer outcomes.^18^ It also is not surprising that women experiencing disparities are more likely to experience greater delay and worsened outcomes. We saw such disparities, where race, insurance status, and education were associated with longer TTS.

We observed that survival differences over time between invasive and noninvasive cohorts remained clinically small in the short term (e.g., approximately 1% at 5 years), consistent with other data where invasive disease is minimal.^26^ However, our findings suggest that invasion is more frequently found as delay to surgery increases, suggesting that long-term delay or nonoperative management may have a significant impact on OS. When grouped by TTS, we observed OS differences varying, with earlier declines in OS observed in women who experienced delays greater than 6 months compared with those with shorter TTS (eFig. 1). The small OS differences seen between Stage 0 pathology and those diagnosed with minimal invasive disease may be explained by the fact that not all invasion is identified on final pathology. This also may be why we see a decline in OS with delay, even for “pure” DCIS. Prior studies reported 0–6% incidence of nodal metastases discovered in the setting of pTis postoperative histology, which is lower than the 13–50% sampling error rate associated with preoperative core needle biopsy, and is attributed to occult invasion.^27^–^33^ Additionally, the number of women who were found to have invasive cancer postoperatively was comparatively small, so the interaction test between delay and pathologic diagnosis of invasion may have had low power. Our survival data suggest that at least some DCIS lesions having no invasive foci identified may be similar to those having small invasive foci identified. This underscores the need to determine definitively which DCIS is truly noninvasive and remains so, posing little risk to the patient.

Questions regarding the extent or overall need for resection of DCIS in North America and Europe have prompted several clinical trials. Designed to clarify whether surgery can be eliminated while solely using endocrine therapy and close observation for low-risk DCIS, the COMET study is an ongoing, phase III, randomized, clinical trial enrolling in the United States.^34^ A similar European investigation randomizing women with DCIS to standard surgical-based care or mammographic observation has begun as the LORIS trial.^35^ The LORD study features a similar clinical design through which women with low-risk disease are randomized to either standard surgical-based treatment or surveillance alone.^36^ Given the debate regarding appropriate treatment of DCIS, the results of these multicenter trials will be extremely valuable. Our data demonstrate that delays to surgery portend worse outcomes, suggesting that nonoperative management may increase risk for DCIS patients when controlling for confounders. Until prospective, randomized data are available, our data do not yet support a change in the standard of care, which involves excision for those who will tolerate it. We therefore advocate nonoperative management solely in the context of clinical trials at this time and advocate for enrollment in those trials because of the association of delay with both invasion and survival.

Our study has several strengths and potential limitations. Our use of a large, contemporary cohort provided sufficient sample size to analyze delays to surgery and outcomes, while maintaining cohort generalizability. Our data were adjusted for facility volume as a surrogate for institutional expertise, which could add unmeasured confounding. Finally, our use of propensity-weighting allowed analysis of time to surgery, pathologic invasiveness, and overall survival in the setting of clinical DCIS, controlling for both characteristics and nonrandom treatment assignment.

Like any retrospective series, our study may be subject to potential biases and unmeasured confounding. Although the NCDB is a well-established quality dataset, data are limited by how well it was coded. Similarly, although phenotype is prognostic and we included grade and receptor status among our covariates, other nonstandard features are not typically included in the NCDB for DCIS diagnoses (e.g., HER2 status) but might be considered when interpreting these data.^37^,^38^ Additionally, changes to ensure de-identification limited inclusion of certain covariates of interest (e.g., NCDB censors facility type and location for all patients < 40 years). Finally, although the NCDB does not include disease-specific survival, OS remains a valid outcome, having the advantage of including competing mortality risks associated with therapy. These observations are important for our study; existing data have shown that while women with DCIS have better OS than the general population, they also have lower breast cancer-specific survival.^39^ The role of these competing risks versus disease-specific mortality will need to be explored through ongoing trials.

## Conclusions

In women with a clinical diagnosis of DCIS, greater delay to surgery is associated with lower OS. Although most women with DCIS undergo surgical extirpation within 2 months of diagnosis, longer time to surgery is associated with greater risk of finding invasion and should be limited. Although clinical trials are underway, this large-scale dataset suggests that delays in nonoperative management of DCIS are associated with invasion and slightly worse short-term outcomes and should not yet be pursued outside of a clinical trial in patients who will tolerate excision. At minimum, these findings also suggest the need for long-term follow-up in women who are observed and better prediction of which subset of DCIS will develop an invasive component.

## Electronic supplementary material

Below is the link to the electronic supplementary material.

Supplementary material 1 (PDF 1267 kb)
