# Supplementary material for: Preoperative Delays in the Treatment of DCIS and the Associated Incidence of Invasive Breast Cancer
Source: Ann Surg Oncol. 2019 Sep 27;27(2):386–96. doi: 10.1245/s10434-019-07844-4 (PMC6949196; doi:10.1245/s10434-019-07844-4)
Supplement: Supplementary file 1 — Supplementary material 1 (PDF 1267 kb) [file 10434_2019_7844_MOESM1_ESM.pdf]

Online Only Content

eMethods: Statistical Methods

eTable 1: Included ICD-O-3 SEER Site/Histology Codes, Breast.

eTable 2: Multivariable Adjusted Associations between Patient Characteristics and Overall Survival

eTable 3: Sensitivity Analysis Examining Association between Patient Characteristics and Overall Survival in Patients with One Surgery Date Recorded (N=101,251).

eTable 4: Adjusted Associations between Characteristics and OS among Postoperative Patients with Non-Invasive Disease.

eTable 5: Adjusted Associations between Characteristics and OS among Postoperative Patients with Invasive Disease.

eTable 6: Multivariable Adjusted Associations between Patient Characteristics and Surgical Delay Grouping.

eTable 7: Point Estimates for 5-Year Overall Survival.

eTable 8: Sensitivity Analysis Examining Association between Patient Characteristics and Invasion in Patients with One Surgery Date Recorded (N=101,251).

eTable 9: Sensitivity Analysis Comparing 5-Year Overall Survival from Time of Diagnosis versus Time of Surgery.

eFigure 1: Overall survival of included DCIS patients grouped by delay interval. CI: confidence interval; DCIS: ductal carcinoma in situ; OS: overall survival.

eFigure 2: Interval length between diagnosis and definitive surgery. Panels represent distribution of patients with noninvasive (top panel) and invasive disease (bottom panel) on final pathology. Each bar represents 1 day.

Statistical Methods

For each cohort, delay groups were compared using Chi-square tests and two sample t-tests. Due to the skewed nature of the data, quantile (median) regressions were used to analyze delays with adjustment for patient, tumor, and treatment characteristics. The bootstrap method with 500 repetitions was used for standard errors. Adjusted median delays were estimated using mean covariate values estimated in the models. Primary survival employed multivariable Cox proportional hazards models, which estimated hazard ratios associated with increasing delay (measured continuously as months) and OS, controlling for relevant covariates measuring patient (age, race, ethnicity, insurance, education, income, urban/rural location, Charlson score, year of diagnosis) , tumor (grade, estrogen receptor status), and treatment (type of surgery, receipt of chemotherapy, receipt of radiotherapy, receipt of endocrine therapy, transfer of care, distance to treatment facility, treating facility volume) factors. In secondary analyses, adjusted survival curves were obtained via inverse probability weighting, where the weights were obtained using generalized propensity scores. Unlike the primary analyses, the propensity score analyses used time until treatment categorized by interval (for ease of computation). Multinomial logistic regression was used to estimate propensity scores with restricted cubic splines for continuous variables, and weighted Kaplan Meier methods were used to estimate 5-year survival rates and to create OS curves. Bootstrap standard errors were used for 95% confidence intervals.

Among all patients, association between delays to surgery and invasion on final pathology was analyzed using the Wilcoxon rank sum test. The Cochran-Armitage trend test was used to analyze trends of invasion by delay group. Predictors of invasion were analyzed using multivariable logistic regression with adjustment for pre-treatment factors. To test whether effect of delay differed by final pathology, interaction terms between delay and invasive pathology were added to the multivariable Cox model.

Statistical significance was set at $p = 0.05$ (two-sided). Analyses were performed using SAS software, version 9.4 (SAS Institute, Cary, NC) and STATA software, release 13 (StataCorp, College Station, TX).

eTable 1: Included ICD-O-3 SEER Site/Histology Codes, Breast

	Site recode	Histology	Histology Description	Histology /Behavior	Histology/Behavior Description
**Ductal Subtypes**	C500-C506,C508-C509	805	PAPILLARY CARCINOMA, NOS	8050/2	Papillary carcinoma in situ
	C500-C506,C508-C509	805	PAPILLARY CARCINOMA, NOS	8050/3	Papillary carcinoma, NOS
	C500-C506,C508-C509	820	ADENOID CYSTIC & CRIBRIFORM CA.	8201/2	Cribriform carcinoma in situ
	C500-C506,C508-C509	820	ADENOID CYSTIC & CRIBRIFORM CA.	8201/3	Cribriform carcinoma
	C500-C506,C508-C509	821	ADENOCA. IN ADENOMA. POLYP	8211/3	Tubular adenocarcinoma
	C500-C506,C508-C509	823	SOLID CARCINOMA, NOS	8230/2	Duct carcinoma in situ, solid type
	C500-C506,C508-C509	823	SOLID CARCINOMA, NOS	8230/3	Solid carcinoma, NOS
	C500-C506,C508-C509	826	PAPILLARY ADENOCARCINOMA, NOS	8260/3	Papillary adenocarcinoma, NOS
	C500-C506,C508-C509	848	MUCINOUS ADENOCARCINOMA	8480/3	Mucinous adenocarcinoma
	C500-C506,C508-C509	848	MUCINOUS ADENOCARCINOMA	8481/3	Mucin-producing adenocarcinoma
	C500-C506,C508-C509	850	DUCT CARCINOMA	8500/2	Intraductal carcinoma, noninfiltrating, NOS
	C500-C506,C508-C509	850	DUCT CARCINOMA	8500/3	Invasive carcinoma of no special type
	C500-C506,C508-C509	850	DUCT CARCINOMA	8501/2	Comedocarcinoma, non-infiltrating
	C500-C506,C508-C509	850	DUCT CARCINOMA	8501/3	Comedocarcinoma, NOS
	C500-C506,C508-C509	850	DUCT CARCINOMA	8502/3	Secretory carcinoma of breast
	C500-C506,C508-C509	850	DUCT CARCINOMA	8503/2	Noninfiltrating intraductal papillary adenocarcinoma
	C500-C506,C508-C509	850	DUCT CARCINOMA	8503/3	Intraductal papillary adenocarcinoma with invasion
	C500-C506,C508-C509	850	DUCT CARCINOMA	8504/2	Noninfiltrating intracystic carcinoma
	C500-C506,C508-C509	850	DUCT CARCINOMA	8504/3	Intracystic carcinoma, NOS

	C500-C506,C508-C509	850	DUCT CARCINOMA	8507/2	Intraductal micropapillary carcinoma
	C500-C506,C508-C509	850	DUCT CARCINOMA	8507/3	Invasive micropapillary carcinoma
	C500-C506,C508-C509	852	LOBULAR AND OTHER DUCTAL CA.	8521/3	Infiltrating ductular carcinoma
	C500-C506,C508-C509	852	LOBULAR AND OTHER DUCTAL CA.	8522/2	Intraductal and lobular in situ carcinoma
	C500-C506,C508-C509	852	LOBULAR AND OTHER DUCTAL CA.	8522/3	Infiltrating duct and lobular carcinoma
	C500-C506,C508-C509	852	LOBULAR AND OTHER DUCTAL CA.	8523/2	Infiltr. duct mixed with other types of carcinoma, in situ
	C500-C506,C508-C509	852	LOBULAR AND OTHER DUCTAL CA.	8523/3	Infiltr. duct mixed with other types of carcinoma
	C500-C506,C508-C509	854	PAGET DISEASE, MAMMARY	8540/3	Paget disease, mammary
	C500-C506,C508-C509	854	PAGET DISEASE, MAMMARY	8541/3	Paget dis. & infil. duct carcinoma
	C500-C506,C508-C509	854	PAGET DISEASE, MAMMARY	8543/3	Paget disease and intraductal ca.
**Lobular Subtypes**	C500-C506,C508-C509	852	LOBULAR AND OTHER DUCTAL CA.	8520/3	Lobular carcinoma, NOS
	C500-C506,C508-C509	852	LOBULAR AND OTHER DUCTAL CA.	8524/3	Infiltrating lobular mixed with other types of carc.
**Other Subtypes**	C500-C506,C508-C509	800	NEOPLASM	8000/3	Neoplasm, malignant
	C500-C506,C508-C509	801	CARCINOMA, NOS	8010/2	Carcinoma in situ, NOS
	C500-C506,C508-C509	801	CARCINOMA, NOS	8010/3	Carcinoma, NOS
	C500-C506,C508-C509	802	CARCINOMA, UNDIFF., NOS	8022/3	Pleomorphic carcinoma
	C500-C506,C508-C509	814	ADENOCARCINOMA, NOS	8140/2	Adenocarcinoma in situ
	C500-C506,C508-C509	814	ADENOCARCINOMA, NOS	8140/3	Adenocarcinoma, NOS
	C500-C506,C508-C509	825	BRONCHIOLO-ALVEOLAR ADENOCA.	8255/3	Adenocarcinoma with mixed subtypes

Adapted from: https://seer.cancer.gov/icd-o-3

eTable 2: Multivariable Adjusted Associations between Patient Characteristics and Overall Survival

Characteristic	N (%)	HR (95%CI)	*P*
**Delay from Diagnosis to Surgery (30-Day Interval) §**	140,615 (100)	1.074 (1.049, 1.099)	< 0.0001
**Age §**	140,615 (100)	1.075 (1.071, 1.079)	< 0.0001
**Race**			
White	115,163 (81.9)	Ref	< 0.0001
Black	17,042 (12.1)	1.088 (1.001, 1.183)	
Asian	5,769 (4.1)	0.578 (0.481, 0.695)	
Other/Unknown	2,641 (1.9)	0.767 (0.597, 0.984)	
**Ethnicity**			
Non-Hispanic	126,904 (90.2)	Ref	< 0.0001
Hispanic	7,016 (5.0)	0.644 (0.549, 0.755)	
Unknown	6,695 (4.8)	1.049 (0.933, 1.180)	
**Insurance**			
Private	87,763 (62.4)	Ref	< 0.0001
Medicaid	6,245 (4.4)	2.025 (1.764, 2.325)	
Medicare	41,365 (29.4)	1.435 (1.333, 1.546)	
Uninsured	2,127 (1.5)	1.737 (1.338, 2.256)	
Government	1,499 (1.1)	1.542 (1.195, 1.990)	
Unknown	1,616 (1.1)	1.457 (1.153, 1.840)	
**Education***			
>21%	17,870 (12.7)	Ref	0.0010
13-20.9%	31,550 (22.4)	0.962 (0.884, 1.048)	
7-12.9%	46,629 (33.2)	0.911 (0.830, 1.000)	
<7%	43,912 (31.2)	0.819 (0.736, 0.911)	
Missing	654 (0.5)	3.790 (0.594, 24.164)	
**Annual Income**			
<$38,000	18,831 (13.4)	Ref	0.0358
$38,000 - $47,999	27,816 (19.8)	0.912 (0.840, 0.991)	
$48,000 - $62,999	36,940 (26.3)	0.907 (0.831, 0.991)	
≥$63,000	56,337 (40.1)	0.852 (0.769, 0.944)	
Missing	691 (0.5)	0.483 (0.088, 2.660)	
**Setting**			
Large Metropolitan	79,489 (56.5)	Ref	0.1744
Small Metropolitan	40,922 (29.1)	1.039 (0.971, 1.111)	
Suburban	10,438 (7.4)	1.056 (0.946, 1.179)	
Rural	5,869 (4.2)	0.920 (0.807, 1.049)	
Unknown	3,897 (2.8)	0.917 (0.754, 1.115)	
**Distance to Treatment Facility (miles)**			
≤10	78,606 (55.9)	Ref	0.1346
11-20	33,342 (23.7)	0.963 (0.903, 1.028)	
21-40	16,577 (11.8)	0.929 (0.850, 1.015)	
>40	11,360 (8.1)	0.884 (0.793, 0.986)	
Unknown	730 (0.5)	1.563 (0.631, 3.872)	
**Transfer of Care**			
No	86,170 (61.3)	Ref	0.0367
Yes	39,602 (28.2)	0.918 (0.859, 0.980)	
Unknown	14,843 (10.6)	0.990 (0.907, 1.082)	
**Treatment Facility Annual Volume**			
0-17 patients (1st Quartile)	8,131 (5.8)	Ref	< 0.0001
18-34 patients (2nd Quartile)	18,054 (12.8)	0.917 (0.816, 1.031)	
35-67 patients (3rd Quartile)	35,074 (24.9)	0.906 (0.813, 1.008)	
>67 patients (4th Quartile)	79,356 (56.4)	0.796 (0.717, 0.883)	
**Year of Diagnosis §**	140,615 (100)	0.992 (0.980, 1.005)	0.2302
**Charlson Comorbidity Score**			
0	120,676 (85.8)	Ref	< 0.0001
1	16,780 (11.9)	1.634 (1.533, 1.742)	
2	2,659 (1.9)	2.813 (2.524, 3.135)	
≥3	500 (0.4)	4.777 (3.859, 5.914)	

**Grade**			
Well Differentiated	17,357 (12.3)	Ref	0.1646
Moderately Differentiated	47,300 (33.6)	1.011 (0.938, 1.090)	
Poorly Differentiated	47,039 (33.5)	1.059 (0.981, 1.143)	
Undifferentiated/Anaplastic	3,684 (2.6)	0.970 (0.837, 1.124)	
Unknown	25,235 (17.9)	0.975 (0.895, 1.062)	
**Surgery Type**			
Breast Conservation	95,326 (67.8)	Ref	< 0.0001
Mastectomy	25,615 (18.2)	0.947 (0.883, 1.015)	
Mastectomy & Reconstruction	27,496 (19.6)	0.623 (0.550, 0.706)	
**Receipt of Chemotherapy**			
No	136,310 (96.9)	Ref	< 0.0001
Yes	4,305 (3.1)	1.902 (1.628, 2.224)	
**Receipt of Radiotherapy**			
No	70,546 (50.2)	Ref	< 0.0001
Yes	70,069 (49.8)	0.700 (0.654, 0.749)	
**Receipt of Endocrine Therapy**			
No	85,881 (61.1)	Ref	< 0.0001
Yes	54,734 (38.9)	0.853 (0.803, 0.906)	
**Estrogen Receptor Status**			
Negative	19,836 (14.1)	Ref	0.0622
Positive	107,305 (76.3)	0.921 (0.859, 0.988)	
Unknown	13,474 (9.6)	0.918 (0.838, 1.004)	

CI, confidence interval; HR, hazard ratio; Ref, reference group; *, percent of adults without a high school diploma by zip code; §, continuous variable.

eTable 3: Sensitivity Analysis Examining Association between Patient Characteristics and Overall Survival in Patients with One Surgery Date Recorded (N=101,251)

Characteristic	HR (95%CI)	*P*
**Delay from Diagnosis to Surgery (30-Day Interval)** §	1.122 (1.087, 1.158)	< 0.0001
**Age** §	1.075 (1.071, 1.080)	< 0.0001
**Race**		
White	Ref	
Black	1.046 (0.945, 1.157)	0.3872
Asian	0.538 (0.441, 0.656)	< 0.0001
Other/Unknown	0.771 (0.594, 1.001)	0.0509
**Ethnicity**		
Non-Hispanic	Ref	
Hispanic	0.649 (0.545, 0.774)	< 0.0001
Unknown	1.068 (0.935, 1.219)	0.3352
**Insurance**		
Private	Ref	
Medicaid	2.212 (1.887, 2.592)	< 0.0001
Medicare	1.430 (1.305, 1.567)	< 0.0001
Uninsured	1.585 (1.160, 2.165)	0.0038
Government	1.463 (1.088, 1.968)	0.0118
Unknown	1.363 (1.010, 1.839)	0.0431
**Education***		
>21%	Ref	
13-20.9%	0.966 (0.876, 1.066)	0.4907
7-12.9%	0.901 (0.810, 1.003)	0.0559
<7%	0.812 (0.718, 0.917)	0.0008
Missing	2.655 (0.361, 19.509)	0.3372
**Annual Income**		
<$38,000	Ref	
$38,000 - $47,999	0.911 (0.826, 1.006)	0.0654
$48,000 - $62,999	0.936 (0.844, 1.038)	0.2103
≥$63,000	0.853 (0.756, 0.964)	0.0107
Missing	0.708 (0.115, 4.343)	0.7088
**Setting**		
Large Metropolitan	Ref	
Small Metropolitan	1.049 (0.969, 1.135)	0.2382
Suburban	1.043 (0.917, 1.185)	0.5224
Rural	0.932 (0.805, 1.080)	0.3519
Unknown	0.930 (0.744, 1.163)	0.5260
**Distance to Treatment Facility (miles)**		
≤10	Ref	
11-20	0.963 (0.891, 1.041)	0.3441
21-40	0.963 (0.867, 1.071)	0.4912
>40	0.916 (0.808, 1.040)	0.1760
Unknown	1.538 (0.554, 4.268)	0.4087
**Transfer of Care**		
No	Ref	
Yes	0.921 (0.854, 0.993)	0.0321
Unknown	0.957 (0.859, 1.066)	0.4221

**Treatment Facility Annual Volume**		
0-17 patients (1st Quartile)	Ref	
18-34 patients (2nd Quartile)	0.914 (0.795, 1.051)	0.2084
35-67 patients (3rd Quartile)	0.914 (0.804, 1.038)	0.1665
>67 patients (4th Quartile)	0.820 (0.724, 0.930)	0.0019
**Year of Diagnosis** §	0.998 (0.984, 1.013)	0.8168
**Charlson Comorbidity Score**		
0	Ref	
1	1.655 (1.535, 1.785)	< 0.0001
2	2.761 (2.429, 3.139)	< 0.0001
≥3	4.898 (3.851, 6.229)	< 0.0001
**Grade**		
Well Differentiated	Ref	
Moderately Differentiated	1.004 (0.919, 1.098)	0.9281
Poorly Differentiated	1.042 (0.952, 1.140)	0.3711
Undifferentiated/Anaplastic	0.908 (0.752, 1.096)	0.3153
Unknown	0.970 (0.877, 1.073)	0.5571
**Surgery Type**		
Breast Conservation	Ref	
Mastectomy	0.933 (0.859, 1.013)	0.1005
Mastectomy & Reconstruction	0.637 (0.548, 0.740)	< 0.0001
**Receipt of Chemotherapy**		
No	Ref	
Yes	1.946 (1.608, 2.355)	< 0.0001
**Receipt of Radiotherapy**		
No	Ref	
Yes	0.687 (0.635, 0.743)	< 0.0001
**Receipt of Endocrine Therapy**		
No	Ref	
Yes	0.846 (0.788, 0.909)	< 0.0001
**Estrogen Receptor Status**		
Negative	Ref	
Positive	0.899 (0.828, 0.975)	0.0105
Unknown	0.896 (0.803, 0.999)	0.0479

CI, confidence interval; HR, hazard ratio; Ref, reference group; *, percent of adults without a high school diploma by zip code; §, continuous variable.

eTable 4: Adjusted Associations between Characteristics and OS among Postoperative Patients with Non-Invasive Disease

Characteristic	HR (95%CI)	*P*
**Delay from Diagnosis to Surgery (30-Day Interval)** §	1.073 (1.047, 1.101)	< 0.0001
**Age** §	1.078 (1.074, 1.081)	< 0.0001
**Race**		
White	Ref	
Black	1.075 (0.985, 1.174)	0.1053
Asian	0.616 (0.505, 0.751)	< 0.0001
Other/Unknown	0.739 (0.566, 0.965)	0.0263
**Ethnicity**		
Non-Hispanic	Ref	
Hispanic	0.655 (0.551, 0.778)	< 0.0001
Unknown	1.064 (0.942, 1.202)	0.3181
**Insurance**		
Private	Ref	
Medicaid	1.935 (1.653, 2.266)	< 0.0001
Medicare	1.405 (1.302, 1.517)	< 0.0001
Uninsured	1.842 (1.386, 2.447)	< 0.0001
Government	1.533 (1.166, 2.017)	0.0022
Unknown	1.456 (1.141, 1.860)	0.0026
**Education***		
>21%	Ref	
13-20.9%	0.981 (0.896, 1.075)	0.6840
7-12.9%	0.932 (0.843, 1.029)	0.1641
<7%	0.829 (0.739, 0.930)	0.0014
Missing	2.616 (0.363, 18.856)	0.3401
**Annual Income**		
<$38,000	Ref	
$38,000 - $47,999	0.901 (0.825, 0.985)	0.0223
$48,000 - $62,999	0.904 (0.822, 0.994)	0.0380
≥$63,000	0.838 (0.751, 0.936)	0.0017
Missing	0.720 (0.116, 4.475)	0.7246
**Setting**		
Large Metropolitan	Ref	
Small Metropolitan	1.025 (0.956, 1.099)	0.4939
Suburban	1.026 (0.911, 1.155)	0.6769
Rural	0.920 (0.799, 1.058)	0.2413
Unknown	0.938 (0.760, 1.156)	0.5465
**Distance to Treatment Facility (miles)**		
≤10	Ref	
11-20	0.976 (0.910, 1.046)	0.4917
21-40	0.936 (0.852, 1.029)	0.1730
>40	0.863 (0.768, 0.970)	0.0138
Unknown	1.576 (0.635, 3.911)	0.3271
**Transfer of Care**		
No	Ref	
Yes	0.910 (0.848, 0.976)	0.0088
Unknown	0.967 (0.879, 1.064)	0.4960

**Treatment Facility Annual Volume**		
0-17 patients (1st Quartile)	Ref	
18-34 patients (2nd Quartile)	0.929 (0.822, 1.051)	0.2441
35-67 patients (3rd Quartile)	0.915 (0.817, 1.025)	0.1247
>67 patients (4th Quartile)	0.805 (0.721, 0.898)	0.0001
**Year of Diagnosis** §	0.994 (0.980, 1.007)	0.3638
**Charlson Comorbidity Score**		
0	Ref	
1	1.635 (1.529, 1.748)	< 0.0001
2	2.930 (2.605, 3.295)	< 0.0001
≥3	4.781 (3.837, 5.957)	< 0.0001
**Grade**		
Well Differentiated	Ref	
Moderately Differentiated	1.049 (0.965, 1.141)	0.2595
Poorly Differentiated	1.094 (1.003, 1.192)	0.0415
Undifferentiated/Anaplastic	0.993 (0.852, 1.158)	0.9301
Unknown	1.024 (0.932, 1.126)	0.6164
**Surgery Type**		
Breast Conservation	Ref	
Mastectomy	0.932 (0.866, 1.002)	0.0569
Mastectomy & Reconstruction	0.576 (0.498, 0.666)	< 0.0001
**Receipt of Chemotherapy**		
No	Ref	
Yes	1.819 (1.347, 2.455)	< 0.0001
**Receipt of Radiotherapy**		
No	Ref	
Yes	0.707 (0.660, 0.759)	< 0.0001
**Receipt of Endocrine Therapy**		
No	Ref	
Yes	0.857 (0.803, 0.914)	< 0.0001
**Estrogen Receptor Status**		
Negative	Ref	
Positive	0.970 (0.897, 1.048)	0.4385
Unknown	0.981 (0.890, 1.082)	0.7075

CI, confidence interval; HR, hazard ratio; OS, overall survival; Ref, reference group; *, percent of adults without a high school diploma by zip code; §, continuous variable.

eTable 5: Adjusted Associations between Characteristics and OS among Postoperative Patients with Invasive Disease

Characteristic	HR (95%CI)	*P*
**Delay from Diagnosis to Surgery (30-Day Interval) §**	1.068 (1.006, 1.134)	0.0306
**Age §**	1.057 (1.047, 1.068)	< 0.0001
**Race**		
White	Ref	
Black	1.150 (0.940, 1.407)	0.1741
Asian	0.358 (0.205, 0.623)	0.0003
Other/Unknown	1.013 (0.571, 1.795)	0.9657
**Ethnicity**		
Non-Hispanic	Ref	
Hispanic	0.554 (0.366, 0.840)	0.0054
Unknown	0.954 (0.689, 1.321)	0.7754
**Insurance**		
Private	Ref	
Medicaid	2.492 (1.840, 3.377)	< 0.0001
Medicare	1.661 (1.336, 2.065)	< 0.0001
Uninsured	1.254 (0.613, 2.565)	0.5357
Government	1.558 (0.786, 3.087)	0.2038
Unknown	1.535 (0.733, 3.215)	0.2559
**Education***		
>21%	Ref	
13-20.9%	0.831 (0.673, 1.025)	0.0836
7-12.9%	0.760 (0.602, 0.959)	0.0207
<7%	0.742 (0.561, 0.981)	0.0360
Missing	8796.560 (1754.674, 44099.050)	< 0.0001
**Annual Income**		
<$38,000	Ref	
$38,000 - $47,999	0.961 (0.773, 1.194)	0.7185
$48,000 - $62,999	0.915 (0.727, 1.151)	0.4460
≥$63,000	0.940 (0.716, 1.234)	0.6545
Missing	0 0 0	< 0.0001
**Setting**		
Large Metropolitan	Ref	
Small Metropolitan	1.144 (0.956, 1.369)	0.1418
Suburban	1.227 (0.942, 1.600)	0.1299
Rural	0.893 (0.619, 1.289)	0.5457
Unknown	0.706 (0.421, 1.183)	0.1865
**Distance to Treatment Facility (miles)**		
≤10	Ref	
11-20	0.888 (0.743, 1.061)	0.1917
21-40	0.909 (0.716, 1.154)	0.4331
>40	0.988 (0.757, 1.292)	0.9323
Unknown	2.776 (1.003, 7.681)	0.0493
**Transfer of Care**		
No	Ref	
Yes	0.926 (0.781, 1.098)	0.3754
Unknown	1.146 (0.891, 1.475)	0.2871

**Treatment Facility Annual Volume**		
0-17 patients (1st Quartile)	Ref	
18-34 patients (2nd Quartile)	0.753 (0.544, 1.044)	0.0891
35-67 patients (3rd Quartile)	0.775 (0.575, 1.045)	0.0951
>67 patients (4th Quartile)	0.646 (0.484, 0.862)	0.0030
**Year of Diagnosis** §	0.979 (0.943, 1.016)	0.2601
**Charlson Comorbidity Score**		
0	Ref	
1	1.638 (1.379, 1.945)	< 0.0001
2	2.104 (1.550, 2.856)	< 0.0001
≥3	4.781 (2.526, 9.050)	< 0.0001
**Grade**		
Well Differentiated	Ref	
Moderately Differentiated	0.875 (0.734, 1.043)	0.1363
Poorly Differentiated	1.098 (0.911, 1.324)	0.3256
Undifferentiated/Anaplastic	1.606 (0.956, 2.696)	0.0733
Unknown	0.794 (0.629, 1.003)	0.0527
**Surgery Type**		
Breast Conservation	Ref	
Mastectomy	0.951 (0.748, 1.208)	0.6792
Mastectomy & Reconstruction	0.727 (0.549, 0.964)	0.0266
**Receipt of Chemotherapy**		
No	Ref	
Yes	1.315 (1.078, 1.604)	0.0068
**Receipt of Radiotherapy**		
No	Ref	
Yes	0.623 (0.486, 0.799)	0.0002
**Receipt of Endocrine Therapy**		
No	Ref	
Yes	0.747 (0.633, 0.881)	0.0005
**Estrogen Receptor Status**		
Negative	Ref	
Positive	0.842 (0.698, 1.016)	0.0720
Unknown	0.688 (0.477, 0.991)	0.0446

CI, confidence interval; HR, hazard ratio; OS, overall survival; Ref, reference group; *, percent of adults without a high school diploma by zip code, §, continuous variable.

eTable 6: Multivariable Adjusted Associations between Patient Characteristics and Surgical Delay Grouping

Time to Surgery	≤30 Days (n=52,816) N (%)	31-60 Days (n=54,680) N (%)	61-90 Days (n=20,600) N (%)	91-120 Days (n=7,326) N (%)	121-180 Days (n=3,899) N (%)	180-240 Days (n=880) N (%)	241-365 Days (n=414) N (%)	Total (n=140,615)	*P*
**Age**									
<50	10844 (20.5)	14132 (25.8)	6460 (31.4)	2403 (32.8)	1364 (35.0)	309 (35.1)	150 (36.2)	35662 (25.4)	<0.001
50-59	14791 (28.0)	15948 (29.2)	6003 (29.1)	2211 (30.2)	1163 (29.8)	263 (29.9)	111 (26.8)	40490 (28.8)	
60-69	15042 (28.5)	14231 (26.0)	4973 (24.1)	1684 (23.0)	870 (22.3)	189 (21.5)	92 (22.2)	37081 (26.4)	
≥70	12139 (23.0)	10369 (19.0)	3164 (15.4)	1028 (14.0)	502 (12.9)	119 (13.5)	61 (14.7)	27382 (19.5)	
**Race**									
White	45274 (85.7)	44851 (82.0)	16034 (77.8)	5421 (74.0)	2738 (70.2)	586 (66.6)	259 (62.6)	115163 (81.9)	<0.001
Black	4885 (9.2)	6556 (12.0)	3106 (15.1)	1339 (18.3)	839 (21.5)	202 (23.0)	115 (27.8)	17042 (12.1)	
Asian	1804 (3.4)	2233 (4.1)	1027 (5.0)	389 (5.3)	222 (5.7)	∂	∂	5769 (4.1)	
Other/Unknown	853 (1.6)	1040 (1.9)	433 (2.1)	177 (2.4)	100 (2.6)	∂	∂	2641 (1.9)	
**Ethnicity**									
Non-Hispanic	48083 (91.0)	49456 (90.4)	18380 (89.2)	6517 (89.0)	3355 (86.0)	759 (86.3)	354 (85.5)	126904 (90.2)	<0.001
Hispanic	1954 (3.7)	2673 (4.9)	1358 (6.6)	527 (7.2)	376 (9.6)	89 (10.1)	39 (9.4)	7016 (5.0)	
Unknown	2779 (5.3)	2551 (4.7)	862 (4.2)	282 (3.8)	168 (4.3)	32 (3.6)	21 (5.1)	6695 (4.8)	
**Insurance**									
Medicaid	1633 (3.1)	2344 (4.3)	1232 (6.0)	549 (7.5)	362 (9.3)	85 (9.7)	40 (9.7)	6245 (4.4)	<0.001
Medicare	17801 (33.7)	15613 (28.6)	5045 (24.5)	1732 (23.6)	851 (21.8)	217 (24.7)	106 (25.6)	41365 (29.4)	
Uninsured	609 (1.2)	774 (1.4)	407 (2.0)	161 (2.2)	129 (3.3)	23 (2.6)	24 (5.8)	2127 (1.5)	
Government	491 (0.9)	596 (1.1)	215 (1.0)	115 (1.6)	57 (1.5)	∂	∂	1499 (1.1)	
Private	31716 (60.0)	34776 (63.6)	13416 (65.1)	4670 (63.7)	2434 (62.4)	523 (59.4)	228 (55.1)	87763 (62.4)	
Unknown	566 (1.1)	577 (1.1)	285 (1.4)	99 (1.4)	66 (1.7)	∂	∂	1616 (1.1)	
**Education***									
>21%	6263 (11.9)	6732 (12.3)	2826 (13.7)	1107 (15.1)	710 (18.2)	∂	∂	17870 (12.7)	<0.001
13-20.9%	12019 (22.8)	12104 (22.1)	4571 (22.2)	1694 (23.1)	829 (21.3)	228 (25.9)	105 (25.4)	31550 (22.4)	
7-12.9%	17849 (33.8)	18150 (33.2)	6726 (32.7)	2313 (31.6)	1212 (31.1)	258 (29.3)	121 (29.2)	46629 (33.2)	
<7%	16403 (31.1)	17461 (31.9)	6401 (31.1)	2179 (29.7)	1129 (29.0)	237 (26.9)	102 (24.6)	43912 (31.2)	
Missing	282 (0.5)	233 (0.4)	76 (0.4)	33 (0.5)	19 (0.5)	∂	∂	654 (0.5)	
**Annual Income**									
<$38,000	7088 (13.4)	7071 (12.9)	2743 (13.3)	1090 (14.9)	617 (15.8)	∂	∂	18831 (13.4)	<0.001
$38,000 - $47,999	11208 (21.2)	10509 (19.2)	3811 (18.5)	1357 (18.5)	683 (17.5)	168 (19.1)	80 (19.3)	27816 (19.8)	
$48,000 - $62,999	14303 (27.1)	14280 (26.1)	5326 (25.9)	1769 (24.1)	939 (24.1)	211 (24.0)	112 (27.1)	36940 (26.3)	

≥$63,000	19918 (37.7)	22577 (41.3)	8637 (41.9)	3077 (42.0)	1638 (42.0)	348 (39.5)	142 (34.3)	56337 (40.1)	
Missing	299 (0.6)	243 (0.4)	83 (0.4)	33 (0.5)	22 (0.6)	∂	∂	691 (0.5)	
**Setting**									
Large Metropolitan	26678 (50.5)	31749 (58.1)	12839 (62.3)	4766 (65.1)	2589 (66.4)	588 (66.8)	280 (67.6)	79489 (56.5)	<0.001
Small Metropolitan	17220 (32.6)	15501 (28.3)	5338 (25.9)	1726 (23.6)	867 (22.2)	187 (21.3)	83 (20.0)	40922 (29.1)	
Suburban	4653 (8.8)	3854 (7.0)	1224 (5.9)	411 (5.6)	223 (5.7)	46 (5.2)	27 (6.5)	10438 (7.4)	
Rural	2842 (5.4)	2059 (3.8)	640 (3.1)	198 (2.7)	97 (2.5)	∂	∂	5869 (4.2)	
Unknown	1423 (2.7)	1517 (2.8)	559 (2.7)	225 (3.1)	123 (3.2)	∂	∂	3897 (2.8)	
**Distance to Treatment Facility (miles)**									
≤10	29924 (56.7)	30308 (55.4)	11384 (55.3)	4098 (55.9)	2181 (55.9)	495 (56.3)	216 (52.2)	78606 (55.9)	<0.001
11-20	12472 (23.6)	13178 (24.1)	4849 (23.5)	1673 (22.8)	885 (22.7)	192 (21.8)	93 (22.5)	33342 (23.7)	
21-40	6291 (11.9)	6514 (11.9)	2357 (11.4)	838 (11.4)	434 (11.1)	∂	∂	16577 (11.8)	
>40	3838 (7.3)	4391 (8.0)	1922 (9.3)	681 (9.3)	384 (9.8)	∂	∂	11360 (8.1)	
Unknown	291 (0.6)	289 (0.5)	88 (0.4)	36 (0.5)	15 (0.4)	∂	∂	730 (0.5)	
**Transfer of Care**									
No	34891 (66.1)	33133 (60.6)	11541 (56.0)	4016 (54.8)	1996 (51.2)	410 (46.6)	183 (44.2)	86170 (61.3)	<0.001
Yes	11409 (21.6)	15950 (29.2)	7275 (35.3)	2720 (37.1)	1638 (42.0)	406 (46.1)	204 (49.3)	39602 (28.2)	
Unknown	6516 (12.3)	5597 (10.2)	1784 (8.7)	590 (8.1)	265 (6.8)	64 (7.3)	27 (6.5)	14843 (10.6)	
**Treatment Facility Annual Volume**									
0-17 patients (1st Quartile)	3813 (7.2)	2913 (5.3)	897 (4.4)	297 (4.1)	160 (4.1)	29 (3.3)	22 (5.3)	8131 (5.8)	<0.001
18-34 patients (2nd Quartile)	8159 (15.4)	6281 (11.5)	2216 (10.8)	852 (11.6)	413 (10.6)	91 (10.3)	42 (10.1)	18054 (12.8)	
35-67 patients (3rd Quartile)	14087 (26.7)	13449 (24.6)	4742 (23.0)	1627 (22.2)	876 (22.5)	191 (21.7)	102 (24.6)	35074 (24.9)	
>67 patients (4th Quartile)	26757 (50.7)	32037 (58.6)	12745 (61.9)	4550 (62.1)	2450 (62.8)	569 (64.7)	248 (59.9)	79356 (56.4)	
**Year of Diagnosis**									
2004	2298 (4.4)	1556 (2.8)	552 (2.7)	203 (2.8)	110 (2.8)	21 (2.4)	18 (4.3)	4758 (3.4)	<0.001
2005	2427 (4.6)	1797 (3.3)	638 (3.1)	245 (3.3)	164 (4.2)	27 (3.1)	20 (4.8)	5318 (3.8)	
2006	2854 (5.4)	2070 (3.8)	738 (3.6)	330 (4.5)	186 (4.8)	41 (4.7)	19 (4.6)	6238 (4.4)	
2007	2994 (5.7)	2740 (5.0)	1015 (4.9)	387 (5.3)	240 (6.2)	43 (4.9)	25 (6.0)	7444 (5.3)	
2008	4831 (9.1)	4610 (8.4)	1777 (8.6)	646 (8.8)	371 (9.5)	84 (9.5)	44 (10.6)	12363 (8.8)	
2009	5602 (10.6)	5693 (10.4)	2155 (10.5)	759 (10.4)	386 (9.9)	87 (9.9)	31 (7.5)	14713 (10.5)	
2010	5955 (11.3)	5979 (10.9)	2257 (11.0)	845 (11.5)	429 (11.0)	114 (13.0)	57 (13.8)	15636 (11.1)	
2011	6293 (11.9)	6848 (12.5)	2374 (11.5)	826 (11.3)	447 (11.5)	108 (12.3)	58 (14.0)	16954 (12.1)	
2012	6521 (12.3)	7336 (13.4)	2867 (13.9)	950 (13.0)	500 (12.8)	102 (11.6)	44 (10.6)	18320 (13.0)	
2013	6679 (12.6)	8038 (14.7)	3132 (15.2)	1066 (14.6)	549 (14.1)	119 (13.5)	43 (10.4)	19626 (14.0)	
2014	6362 (12.0)	8013 (14.7)	3095 (15.0)	1069 (14.6)	517 (13.3)	134 (15.2)	55 (13.3)	19245 (13.7)	
**Charlson Comorbidity Score**									

0	45482 (86.1)	46939 (85.8)	17621 (85.5)	6233 (85.1)	3299 (84.6)	756 (85.9)	346 (83.6)	120676 (85.8)	0.001
1	6250 (11.8)	6501 (11.9)	2470 (12.0)	901 (12.3)	503 (12.9)	104 (11.8)	51 (12.3)	16780 (11.9)	
2	928 (1.8)	1043 (1.9)	416 (2.0)	158 (2.2)	84 (2.2)	∂	∂	2659 (1.9)	
≥3	156 (0.3)	197 (0.4)	93 (0.5)	34 (0.5)	13 (0.3)	∂	∂	500 (0.4)	
**Grade**									
Well Differentiated	6800 (12.9)	6649 (12.2)	2367 (11.5)	895 (12.2)	469 (12.0)	∂	∂	17357 (12.3)	<0.001
Moderately Differentiated	17226 (32.6)	18453 (33.7)	7192 (34.9)	2554 (34.9)	1410 (36.2)	311 (35.3)	154 (37.2)	47300 (33.6)	
Poorly Differentiated	17543 (33.2)	18542 (33.9)	6921 (33.6)	2396 (32.7)	1258 (32.3)	265 (30.1)	114 (27.5)	47039 (33.5)	
Undifferentiated/Anaplastic	1426 (2.7)	1376 (2.5)	547 (2.7)	200 (2.7)	103 (2.6)	∂	∂	3684 (2.6)	
Unknown	9821 (18.6)	9660 (17.7)	3573 (17.3)	1281 (17.5)	659 (16.9)	162 (18.4)	79 (19.1)	25235 (17.9)	
**Surgery Type**									
Breast Conservation	40934 (77.5)	33857 (61.9)	9735 (47.3)	2925 (39.9)	1292 (33.1)	260 (29.5)	153 (37.0)	89156 (63.4)	<0.001
Mastectomy	7865 (14.9)	9576 (17.5)	4262 (20.7)	1736 (23.7)	1070 (27.4)	268 (30.5)	128 (30.9)	24905 (17.7)	
Mastectomy & Reconstruction	4017 (7.6)	11247 (20.6)	6603 (32.1)	2665 (36.4)	1537 (39.4)	352 (40.0)	133 (32.1)	26554 (18.9)	
**Receipt of Chemotherapy**									
Yes	1309 (2.5)	1827 (3.3)	767 (3.7)	254 (3.5)	121 (3.1)	∂	∂	4305 (3.1)	<0.001
No	51507 (97.5)	52853 (96.7)	19833 (96.3)	7072 (96.5)	3778 (96.9)	∂	∂	136310 (96.9)	
**Receipt of Radiotherapy**									
Yes	32175 (60.9)	26970 (49.3)	7619 (37.0)	2168 (29.6)	888 (22.8)	160 (18.2)	89 (21.5)	70069 (49.8)	<0.001
No	20641 (39.1)	27710 (50.7)	12981 (63.0)	5158 (70.4)	3011 (77.2)	720 (81.8)	325 (78.5)	70546 (50.2)	
**Receipt of Endocrine Therapy**									
Yes	22734 (43.0)	21242 (38.8)	7131 (34.6)	2314 (31.6)	1030 (26.4)	199 (22.6)	84 (20.3)	54734 (38.9)	<0.001
No	30082 (57.0)	33438 (61.2)	13469 (65.4)	5012 (68.4)	2869 (73.6)	681 (77.4)	330 (79.7)	85881 (61.1)	
**Tumor Size‡**									
<10mm	2895 (60.3)	4135 (60.6)	1896 (61.4)	696 (61.4)	397 (62.4)	76 (61.8)	28 (47.5)	10123 (60.7)	0.003
10-19mm	740 (15.4)	1083 (15.9)	475 (15.4)	194 (17.1)	84 (13.2)	∂	∂	2609 (15.7)	
≥20mm	373 (7.8)	482 (7.1)	213 (6.9)	61 (5.4)	63 (9.9)	∂	∂	1207 (7.2)	
Unknown	792 (16.5)	1127 (16.5)	505 (16.3)	183 (16.1)	92 (14.5)	∂	∂	2729 (16.4)	
**Pathologic Stage**									
0	48016 (90.9)	47853 (87.5)	17511 (85.0)	6192 (84.5)	3263 (83.7)	757 (86.0)	355 (85.7)	123947 (88.1)	<0.001
1	4183 (7.9)	5952 (10.9)	2670 (13.0)	997 (13.6)	552 (14.2)	107 (12.2)	46 (11.1)	14507 (10.3)	
2	521 (1.0)	733 (1.3)	365 (1.8)	108 (1.5)	67 (1.7)	∂	∂	1818 (1.3)	
3	∂	∂	∂	∂	∂	∂	∂	326 (0.2)	
4	∂	∂	∂	∂	∂	∂	∂	17 (0.0)	
**Estrogen Receptor Status**									
Negative	7353 (13.9)	7777 (14.2)	2974 (14.4)	1006 (13.7)	560 (14.4)	110 (12.5)	56 (13.5)	19836 (14.1)	<0.001
Positive	39872 (75.5)	42061 (76.9)	15811 (76.8)	5633 (76.9)	2953 (75.7)	672 (76.4)	303 (73.2)	107305 (76.3)	

Unknown	5591 (10.6)	4842 (8.9)	1815 (8.8)	687 (9.4)	386 (9.9)	98 (11.1)	55 (13.3)	13474 (9.6)	

*, percent of adults without a high school diploma by zip code; ‡, patients with invasive disease only; ∂, as per NCDB requirements, cells containing <11 individuals and any cells making them calculable have been censored.

eTable 7: Point Estimates for 5-Year Overall Survival

Cohort	Notes	OS Point Estimates
All Patients	Unadjusted	95.8%
By Invasion		
No Invasion	Model-based, averaged over all covariates, including delay	96.0%
Invasion	Model-based, averaged over all covariates, including delay	94.9%
By Delay		
TTS 0 months	Model-based, averaged over all covariates, including invasion but excluding delay	96.3%
TTS 3 months	Model-based, averaged over all covariates, including invasion but excluding delay	95.4%
TTS 6 months	Model-based, averaged over all covariates, including invasion but excluding delay	94.4%

OS, overall survival; TTS, time to surgery.

eTable 8: Sensitivity Analysis Examining Association between Patient Characteristics and Invasion in Patients with One Surgery Date Recorded (N=101,251)

Characteristic	OR (95%CI)	*P*
**Delay from Diagnosis to Surgery (30-Day Interval)** §	1.120 (1.095, 1.146)	< 0.001
**Age (years)**		
<50	Ref	
50-59	0.752 (0.716, 0.791)	< 0.001
60-69	0.696 (0.655, 0.740)	< 0.001
≥70	0.672 (0.622, 0.727)	< 0.001
**Race**		
White	Ref	
Black	1.034 (0.960, 1.114)	0.375
Asian	1.023 (0.910, 1.150)	0.699
Other/Unknown	0.804 (0.676, 0.957)	0.014
**Ethnicity**		
Non-Hispanic	Ref	
Hispanic	1.061 (0.961, 1.171)	0.242
Unknown	0.897 (0.777, 1.035)	0.137
**Insurance**		
Private	Ref	
Medicaid	1.205 (1.097, 1.323)	< 0.001
Medicare	0.964 (0.902, 1.030)	0.274
Uninsured	1.129 (0.961, 1.328)	0.141
Government	0.979 (0.808, 1.186)	0.830
Unknown	0.738 (0.547, 0.996)	0.047
**Education***		
>21%	Ref	
13-20.9%	0.959 (0.879, 1.045)	0.339
7-12.9%	1.023 (0.929, 1.126)	0.648
<7%	1.070 (0.958, 1.196)	0.230
Missing	0.489 (0.148, 1.613)	0.240
**Annual Income**		
<$38,000	Ref	
$38,000 - $47,999	1.113 (1.019, 1.215)	0.018
$48,000 - $62,999	1.121 (1.016, 1.237)	0.023
≥$63,000	1.083 (0.962, 1.220)	0.186
Missing	2.493 (0.971, 6.399)	0.058
**Setting**		
Large Metropolitan	Ref	
Small Metropolitan	0.894 (0.817, 0.978)	0.015
Suburban	0.970 (0.858, 1.098)	0.634
Rural	0.888 (0.775, 1.018)	0.089
Unknown	1.120 (0.969, 1.294)	0.125
**Distance to Treatment Facility (miles)**		
≤10	Ref	
11-20	1.014 (0.962, 1.069)	0.600
21-40	1.143 (1.050, 1.244)	0.002
>40	1.326 (1.195, 1.471)	< 0.001
Unknown	0.638 (0.295, 1.379)	0.253
**Transfer of Care**		

No	Ref	
Yes	1.214 (1.139, 1.295)	< 0.001
Unknown	1.077 (0.984, 1.178)	0.107
**Treatment Facility Annual Volume**		
0-17 patients (1st Quartile)	Ref	
18-34 patients (2nd Quartile)	1.045 (0.903, 1.209)	0.554
35-67 patients (3rd Quartile)	1.148 (0.993, 1.327)	0.063
>67 patients (4th Quartile)	1.302 (1.133, 1.496)	< 0.001
**Year of Diagnosis** §	1.041 (1.028, 1.055)	< 0.001
**Charlson Comorbidity Score**		
0	Ref	
1	1.127 (1.058, 1.201)	< 0.001
2	1.308 (1.138, 1.503)	< 0.001
≥3	1.178 (0.844, 1.644)	0.335
**Grade**		
Well Differentiated	Ref	
Moderately Differentiated	0.535 (0.500, 0.572)	< 0.001
Poorly Differentiated	0.253 (0.233, 0.274)	< 0.001
Undifferentiated/Anaplastic	0.085 (0.066, 0.111)	< 0.001
Unknown	0.338 (0.290, 0.394)	< 0.001
**Estrogen Receptor Status**		
Negative	Ref	
Positive	0.375 (0.353, 0.398)	< 0.001
Unknown	0.108 (0.093, 0.126)	< 0.001

CI, confidence interval; OR, odds ratio; Ref, reference group; *, percent of adults without a high school diploma by zip code; §, continuous variable.

eTable 9: Sensitivity Analysis Comparing 5-Year Overall Survival from Time of Diagnosis versus Time of Surgery

**Time to Surgery (days)**	**OS as Time from Surgery**	**OS as Time from Diagnosis**	**Analysis of Maximum Likelihood Estimates**					
			Time from Surgery Parameter Estimate	Time from Surgery Standard Error	*P*	Time from Diagnosis Parameter Estimate	Time from Diagnosis Standard Error	*P*
1-30	0.9610	0.9607						
31-60	0.9576	0.9604	0.03678	0.01723	0.0328	0.02025	0.01722	0.2397
61-90	0.9544	0.9576	0.12031	0.01697	<0.0001	0.08179	0.01697	<0.0001
91-120	0.9540	0.9547	0.27774	0.01655	<0.0001	0.21588	0.01654	<0.0001
121-365	0.9365	0.9437	0.40979	0.01616	<0.0001	0.30257	0.01615	<0.0001

OS, overall survival.

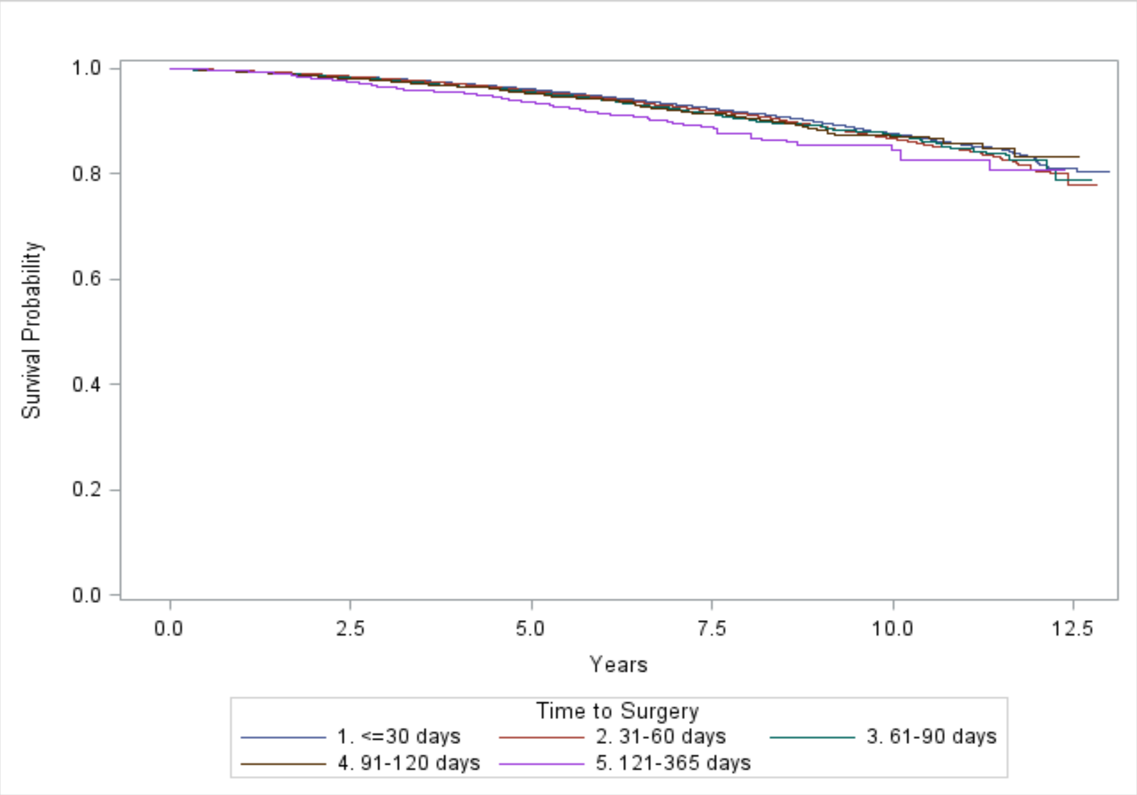

CI: confidence interval; DCIS: ductal carcinoma in situ; OS: overall survival.

Time to Surgery (days)	5-Year OS Probability	95% CI
0-30	0.9610	0.95706, 0.96157
31-60	0.9576	0.95356, 0.95784
61-90	0.9544	0.94719, 0.95542
91-120	0.9540	0.93953, 0.95515
121-365	0.9365	0.90834, 0.93619

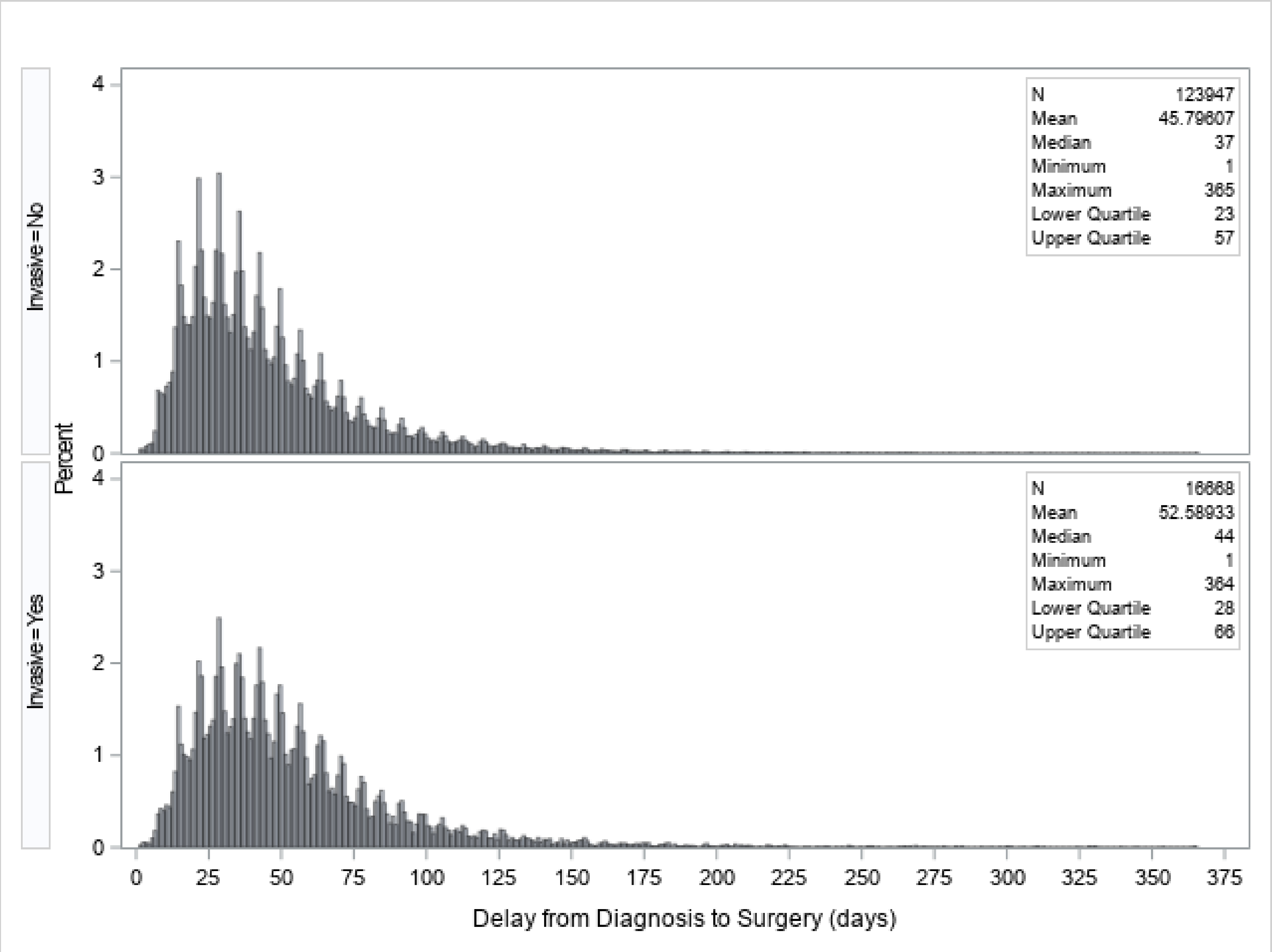

Invasive=No
Invasive=Yes
Percent
N 123947
Mean 45.79607
Median 37
Minimum 1
Maximum 365
Lower Quartile 23
Upper Quartile 57
N 16668
Mean 52.58933
Median 44
Minimum 1
Maximum 364
Lower Quartile 28
Upper Quartile 66
0 1 2 3 4
0 25 50 75 100 125 150 175 200 225 250 275 300 325 350 375
Delay from Diagnosis to Surgery (days)
